# Genome-wide identification of tea plant (*Camellia sinensis*) *BAHD* acyltransferases reveals their role in response to herbivorous pests

**DOI:** 10.1186/s12870-024-04867-2

**Published:** 2024-04-01

**Authors:** Dahe Qiao, Chun Yang, Xiaozeng Mi, Mengsha Tang, Sihui Liang, Zhengwu Chen

**Affiliations:** 1 Guizhou Tea Research Institute, Guizhou Academy of Agricultural Sciences, Guiyang, 550006 Guizhou China; 2 Key Laboratory of Crop Genetic Resources and Germplasm Innovation in Karst Region, Ministry of Agriculture and Rural Affairs, Guizhou Academy of Agricultural Sciences, Guiyang, 550006 Guizhou China

**Keywords:** *Camellia sinensis*, Acyltransferase, Tea geometrid, Tea green leafhopper, HIPVs

## Abstract

**Background:**

BAHD acyltransferases are among the largest metabolic protein domain families in the genomes of terrestrial plants and play important roles in plant growth and development, aroma formation, and biotic and abiotic stress responses. Little is known about the BAHDs in the tea plant, a cash crop rich in secondary metabolites.

**Results:**

In this study, 112 *BAHD* genes (*CsBAHD01*-*CsBAHD112*) were identified from the tea plant genome, with 85% (98/112) unevenly distributed across the 15 chromosomes. The number of BAHD gene family members has significantly expanded from wild tea plants to the *assamica* type to the *sinensis* type. Phylogenetic analysis showed that they could be classified into seven subgroups. Promoter *cis*-acting element analysis revealed that they contain a large number of light, phytohormones, and stress-responsive elements. Many members displayed tissue-specific expression patterns. *CsBAHD05* was expressed at more than 500-fold higher levels in purple tea leaves than in green tea leaves. The genes exhibiting the most significant response to MeJA treatment and feeding by herbivorous pests were primarily concentrated in subgroups 5 and 6. The expression of 23 members of these two subgroups at different time points after feeding by tea green leafhoppers and tea geometrids was examined via qPCR, and the results revealed that the expression of *CsBAHD93*, *CsBAHD94* and *CsBAHD95* was significantly induced after the tea plants were subjected to feeding by both pricking and chewing pests. Moreover, based on the transcriptome data for tea plants being fed on by these two pests, a transcriptional regulatory network of different transcription factor genes coexpressed with these 23 members was constructed.

**Conclusions:**

Our study provides new insights into the role of BAHDs in the defense response of tea plants, and will facilitate in-depth studies of the molecular function of BAHDs in resistance to herbivorous pests.

**Supplementary information:**

The online version contains supplementary material available at 10.1186/s12870-024-04867-2.

## Background

Plants are rich in secondary metabolites, which are essential for plant growth and development, flavor formation, plant-to-plant communication and interactions between plants and the surrounding environment [1]. These functions often require modification by different enzymes, such as glycosylation, methylation and acylation, which further increase the diversity of plant secondary metabolites [2]. In plants, most acylation is catalyzed primarily by serine carboxypeptidase-like acyltransferases (SCPLs) and BAHD acyltransferases, the latter of which constitute one of the 15 largest families of metabolic protein structural domains in the genomes of terrestrial plants, indicating their functional importance in plants [3, 4]. The name of the BAHD acyltransferase family is based on the initials of the first four family members identified in different plants: benzyl alcohol *O*-acetyltransferase (BEAT) from *Clarkia breweri*; anthocyanin *O*-hydroxycinnamoyltransferase (AHCT) from *Petunia*, *Senecio*, *Gentiana*, *Perilla*, and *Lavandula*; anthranilate N-hydroxycinnamoyl/benzoyltransferase (HCBT) from *Dianthus caryophyllus*; and deacetylvindoline 4-*O*-acetyltransferase (DAT) from *Catharanthus roseus* [2, 5]. Although all members of the BAHD family share the PF02458 protein family (Pfam) domain, the sequence similarity between different members is only 10–30% [3, 6], signaling their functional diversity.

With the increasing number of sequenced plant genomes, a large number of BAHDs have been identified from different plants [6–10]. The number of BAHDs has significantly expanded during plant evolution [3]. In angiosperms, phylogenetic analysis based on amino acid sequences classified the BAHD family into seven subgroups (Clades 1–7). Based on the reported catalytic activities, 10 main types of substrates are available for BAHDs, including aromatic alcohols, aliphatic alcohols, aromatic amines, aliphatic amines, terpenoids, flavonoids, anthocyanins, phenolic glucosides, sugar derivatives and alkaloids [3]. The different subgroup members primarily utilized different substrates [3]. Due to the important biological functions of these substrates and their products, an ever-increasing amount of research has focused on the functional resolution of BAHDs in plants. Extensive accumulating evidences have demonstrated that the BAHD family is involved in a wide variety of biological processes, including but not limited to plant growth and development [11, 12], fruit ripening [6, 13], floral formation [14, 15], and biotic and abiotic stress responses [16–19].

The tea plant (*Camellia sinensis*) is an evergreen woody cash crop that originated in Southwest China, and its leaves are the raw materials for making tea, one of the world’s top three nonalcoholic beverages [20]. A warm and humid growing environment makes tea plants highly susceptible to pathogenic bacteria, fungi, and herbivorous pests, and these types of biotic stresses have a serious impact on the yield and quality of tea [21]. Preventing the loss of tea production caused by biotic stress is an urgent problem. Previous studies have demonstrated that it is feasible to utilize tea plants’ own secondary metabolites for the prevention and control of pests and diseases in tea plantations [22–25], and many functional genes involved in the synthesis of these secondary metabolites have been cloned [26–29], which provides important targets for the molecular breeding of tea plants. Notably, many defense-related secondary metabolites such as flavonoids, anthocyanins, alkaloids, aromatic alcohols, aliphatic alcohols, and terpenoids, in tea plants can act as substrates or products of the BAHD family, which indicates that members of the BAHD family may play a key role in the defense response of tea plants. However, to date, there has been little known about the BAHD family in tea plants. In a previous study, 21 BAHD members were identified from the tea plant draft genome, and ectopic overexpression analysis of one member (TEA031065) showed a dramatic increase in growth and anthocyanin content in the transgenic lines [30]. In another study, we found that the expression of the *CsCHAT1* gene was upregulated several hundred-fold after tea plants were subjected to feeding by tea green leafhoppers [31]. Acetyl CoA:(Z)-3-hexen-1-ol acetyltransferase (CHAT) is a member of the BAHD family that catalyzes the formation of (Z)-3-hexen-1-yl acetate from (Z)-3-hexen-1-ol [16], and both of these volatiles can induce plant defense responses [32, 33]. Therefore, further exploration of the role of BAHDs in the defense response in tea plants is necessary.

In this study, we performed a genome-wide systematic identification of the BAHD acyltransferase gene family in tea plants, clarified the distribution characteristics of the genes on the chromosomes of tea plants and the collinearity characteristics of different types of tea plants, and analyzed their sequence characteristics and phylogenetic characteristics. Based on the transcriptome data, the expression patterns of each member in different tissues, in different months, in different leaf colors, and after low-temperature acclimation, MeJA treatment, and tea green leafhoppers feeding were analyzed. Finally, we focused on the members of the core subgroups of the BAHD family in tea plants, and used qPCR to analyze their response characteristics at different time points after feeding by a chewing pest (*Ectropis obliqua*) or a sucking pest (*Empoasca onukii* Matsuda). Based on this, candidate pest response members were screened out, and a pest response transcriptional regulatory network was constructed. Our study provides new insights into the role of BAHDs in the defense response of tea plants, and will facilitate in-depth studies of the molecular function of BAHDs in resistance to herbivorous pests.

## Materials and methods

### Identification of the BAHD acyltransferase family in tea plants

To identify the putative *BAHD* genes in tea plants, the chromosome-level reference genome sequence of *Camellia sinensis* var. *sinensis* cv. ‘Shuchazao’ (SCZ) [34] was first downloaded from the TPIA database (http://tpia.teaplants.cn/) [35]. The reported BAHD protein [2] amino acid sequences of different plants were downloaded from NCBI according to their accession numbers, and subsequently used as queries in local BLASTp searches (E-value ≤ 10^− 10^) against the tea plant annotated protein database. The candidate protein sequences were first filtered out if they were less than 100 amino acids in length, and then further identified using Pfam (https://www.ebi.ac.uk/Tools/pfa/pfamscan/), CDD (https://www.ncbi.nlm.nih.gov/Structure/bwrpsb/bwrpsb.cgi) and SMART (https://smart.embl.de/), and the members containing the PF02458 domain were subjected to subsequent analysis.

### Chromosome distribution and synteny analysis

The GFF files of the identified *BAHD* genes were used to visualize their distribution on the tea plant chromosomes via TBtools software [36], and they were subsequently renamed according to the order of their distribution on the chromosomes. Genome-wide synteny analyses of SCZ, SCZ and LJ43 (*C. sinensis* cv. ‘Longjing43’) [37]; SCZ and TGY (*C. sinensis* cv. ‘Tieguanyin’) [38]; SCZ and YK10 (*C.assamica* cv. ‘Yunkang10’, http://teabase.ynau.edu.cn/index/download/index); and SCZ and DASZ (wild tea plant) [39] were performed using the “One Step MCScanX” plugin of TBtools software and visualized by the “Advanced Circos” or “Dual Systeny Plot for MCScanX” plugins [36].

### Calculating Ka and Ks

The nonsynonymous substitution rate (Ka) and synonymous substitution rate (Ks) of duplicated gene pairs in the *BAHD* gene family of tea plants were estimated using TBtools software. The formula T = Ks/2λ × 10^− 6^ Mya was used to calculate the timing of duplication events as divergence time (T) in millions of years (Mya), where λ = 6.5 × 10^− 9^ represents the rate of replacement of each site per year [40].

### Sequence characteristics of the tea plant *BAHD* gene family

The isoelectric points (pIs), molecular weights (MWs) and grand average hydropathicity (GRAVY) values of the tea plant BAHD proteins were predicted using the online tool on the ExPASy server (https://web.expasy.org/protparam/). The subcellular localization of each protein was predicted using WoLF PSORT (https://wolfpsort.hgc.jp/). The conserved motifs of each protein were predicted using the Multiple Em for Motif Elicitation (MEME) online tool (http://meme-suite.org/tools/meme) based on the default parameters, and the maximum motif number was set to 10. The conserved motifs were subsequently visualized with TBtools software. The gene structures of introns and exons were also visualized by TBtools software through the use of the GFF file of the genome annotation. The 2 kb upstream sequence of the start codon of each gene was considered the promoter region, and the presumed *cis*-regulatory elements of each promoter were analyzed using the PlantCARE online tool (http://bioinformatics.psb.ugent.be/webtools/plantcare/html/).

### Phylogenic analysis

The BAHD sequences of different plants were downloaded from NCBI based on the accession numbers. Then, together with the BAHD family members of the tea plant, the complete amino acid sequences of them were aligned using MUSCLE software, a phylogenetic tree was constructed via IQ-TREE2 software using the maximum likelihood (ML) method, and the bootstrap replicates value were set to 1000. The phylogenetic tree was visualized using the iTOL online tool (https://itol.embl.de/).

### Expression pattern analysis based on the transcriptome data

The public RNA-seq data for eight representative tissues of the plants [40], tea leaves during five different months [41], purple and green tea leaves (PRJNA528853), and plants subjected to cold adaptation [42], exogenous MeJA treatment [43] and tea geometrid feeding [44] were downloaded from the Sequence Read Archive (SRA) database of NCBI. The transcriptomic data for tea plants in response to feeding by tea green leafhoppers were our previously reported [31]. The transcriptome data analysis and gene expression calculation methods have been described previously [45]. The fold difference in gene expression was equal to the FPKM value under treatment plus 1 divided by the FPKM value of the control plus 1. Gene expression heatmaps were drawn using TBtools software.

### Plant materials and tea geometrid and tea green leafhopper feeding treatments

Two-year-old potted seedlings of *Camellia sinensis* cv. ‘Qiancha 1’ (three plants per pot) grown in the tea germplasm resource nursery of the Guizhou Tea Research Institute (26°30′ N, 106°39′ E) were used as materials. The tea geometrid (*Ectropis obliqua*) and tea green leafhopper (*Empoasca onukii* Matsuda) feeding treatments have been described in our previous studies [31, 46]. In brief, for tea geometrid feeding treatment, three-instar larvae were placed evenly on the young leaves of the tea seedlings after starvation for 2 h, and 20 insects were placed in each pot of tea seedlings. When approximately 1/3 of the single leaf was eaten, the insects were removed, and the samples from three biological replicates (three leaves as one biological repeat) were collected at 3, 6, 9, 12, and 24 h after the feeding treatment (E3, E6, E9, E12, and E24). Seedlings without insects were used as controls (CK3, CK6, CK9, CK12, and CK24). For the tea green leafhopper feeding treatment, healthy potted seedlings were moved to the greenhouse, and 24 pots were placed in a 2 m × 1 m × 1 m dense net shed. Approximately 200 adults of *E. onukii* were placed into the shed for treatment. In another room, under the same conditions as those used for the tea seedlings, tea green leafhoppers were not used as a control. When the leaves showed signs of *E. onukii* infection (conspicuous brown spots that appeared near the middle vein), the dense nets and *E. onukii* were removed. Samples were taken from the damaged young leaves at 6, 24, and 48 h (M6, M24, and M48) after removal, and the control samples were taken simultaneously (CK6, CK24, and CK48). At least three tea plant samples were obtained from each replicate, and three repetitions were performed at each time point.

### RNA extraction and quantitative real-time PCR (qRT-PCR) analysis

The total RNAs were extracted using the TaKaRa MiniBEST Plant RNA Extraction Kit (TaKaRa, Japan), and first-strand cDNA synthesis was performed using the PrimeScript RT Reagent Kit (TaKaRa, Japan) according to the manufacturer’s instructions. The primers used were designed with Primer Premier 5 and synthesized by Sangon Biotech (Shanghai, China). The TB Green Premix Ex Taq™ II (TaKaRa, Japan) was used for qRT-PCR on a Bio-Rad CFX96 Real-Time PCR System (Bio-Rad, USA). The reaction system, reaction conditions and internal reference gene selection for qPCR were described in our previous report [31]. The primers used in this study are listed in Table S1.

### Statistical analysis

The statistical analysis of gene expression differences by qRT-PCR was performed using t-tests with GraphPad Prism 9 software (*P < 0.05, and **P < 0.01). All the values presented in the figures are shown as the means ± standard deviations (SDs) of three biological triplicates. The Pearson correlation of genes associated with the expression of different transcription factors was analyzed using the cor() function in R. The gene correlation expression networks were constructed using *P* < 0.01 as a screening parameter and visualized using Cytoscape v3.7.1 software.

## Results

Genome-wide identification and chromosomal locations of *CsBAHDs* in tea plants.

In the tea plant (*C. sinensis* cv. ‘Shuchazao’) genome, a total of 112 sequences were retrieved through a homology search using amino acid sequences of BAHDs from different plants. All of them contained the PF02458 transferase superfamily conserved domain. They were distributed on all 15 chromosomes of the tea plant genome, with the largest distribution on chromosomes 2 and 6, each having 16 and 13, respectively. Based on their chromosomal distribution, the genes were renamed sequentially as *CsBAHD01* to *CsBAHD112*. Among them, *CsBAHD13*-*18*, *CsBAHD32*-*37*, *CsBAHD77*-*81* and *CsBAHD91*-*95* were all distributed in clusters on the chromosomes. Several characteristic parameters of the *CsBAHDs* are listed in Table S2, including their coding sequence length, protein amino acid sequence length, calculated MW, pI and GRAVY. The GRAVY values of most CsBAHDs (102/112) were less than 0, indicating that most of them are hydrophilic proteins. Subcellular localization predictions revealed that the majority of the CsBAHDs are localized in the cytoplasm (56/112) or chloroplasts (33/112), while other members may localize to the nucleus (12/112), mitochondria (CsBAHD25 and CsBAHD95), cytoskeleton (CsBAHD19, CsBAHD77, CsBAHD84 and CsBAHD101), or peroxisomes (CsBAHD40, CsBAHD41, CsBAHD45, CsBAHD46 and CsBAHD55).

### Synteny analysis of *CsBAHDs*

**Fig. 1:**
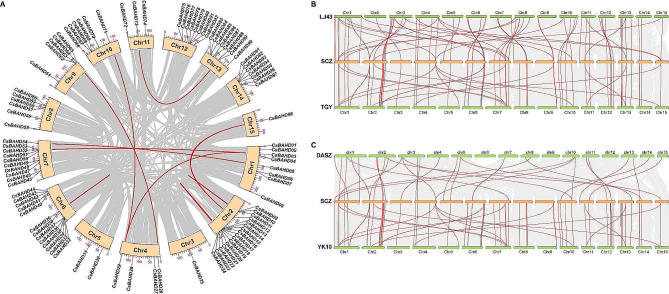
Collinearity of *CsBAHD* gene pairs. (**A**) Collinearity analysis of the *CsBAHD* gene family. The identified *CsBAHD* gene pairs are connected by red lines. (**B**) and (**C**) Collinearity analysis of *CsBAHD* genes between SCZ and LJ43, SCZ and TGY, SCZ and YK10, and SCZ and DASZ. The collinear gene pairs are connected by red lines, and their details are shown in Table S3

The evolutionary characteristics of the *BAHD* gene family in tea plants were explored via synteny analysis. Four gene duplication patterns were identified, including whole-genome duplication (WGD) or segmental duplication, tandem duplication (TD), proximal duplication (PD) and dispersed duplication (DSD), each with 20, 27, 25, and 39 members, respectively (Table S2). In addition, a total of 7 pairs of segmental duplication genes were found within the SCZ genome (Fig. 1A). Among them, *CsBAHD3* and *CsBAHD29* had two duplicate gene pairs distributed on different chromosomes. The Ka/Ks values of the 7 duplicated *BAHD* gene pairs were further analyzed (Table 1). All of the Ka/Ks ratios were less than 1, indicating that they all underwent purifying selection during evolution. The divergence time calculations showed that these genes differentiated between approximately 5.769 Mya (*CsBAHD09*/*CsBAHD10*) and 112 Mya (*CsBAHD03*/*CsBAHD29*). To further investigate the evolution of the *BAHD* gene family in tea plants, collinearity analyses of SCZ and LJ43, SCZ and TGY, SCZ and YK10, and SCZ and DASZ were also conducted. A total of 84, 83, 55 and 35 syntenic gene pairs were identified between the four genome pairs mentioned above (Fig. 1B, C; Table S3). This finding suggested that a significant increase in the number of *BAHD* gene family members occurred from wild tea plants to the *assamica* type to the *sinensis* type.

**Table 1:** The Ka/Ks ratios and date of duplication for duplicate *BAHD* genes in tea plant

Duplicated gene pairs	Ka	Ks	Ka/Ks	Selective pressure	Duplication time (Mya)	Duplicate type
CsBAHD03/CsBAHD29	0.259	1.456	0.178	Purify selection	112.000	Segmental
CsBAHD03/CsBAHD52	0.146	0.833	0.175	Purify selection	64.077	Segmental
CsBAHD05/CsBAHD54	0.133	0.991	0.135	Purify selection	76.231	Segmental
CsBAHD09/CsBAHD10	0.039	0.075	0.518	Purify selection	5.769	Segmental
CsBAHD98/CsBAHD12	0.104	1.056	0.098	Purify selection	81.231	Segmental
CsBAHD71/CsBAHD29	0.114	0.724	0.158	Purify selection	55.692	Segmental
CsBAHD73/CsBAHD88	0.094	0.474	0.199	Purify selection	36.462	Segmental

### Phylogenetic analysis and classification of CsBAHDs

**Fig. 2:**
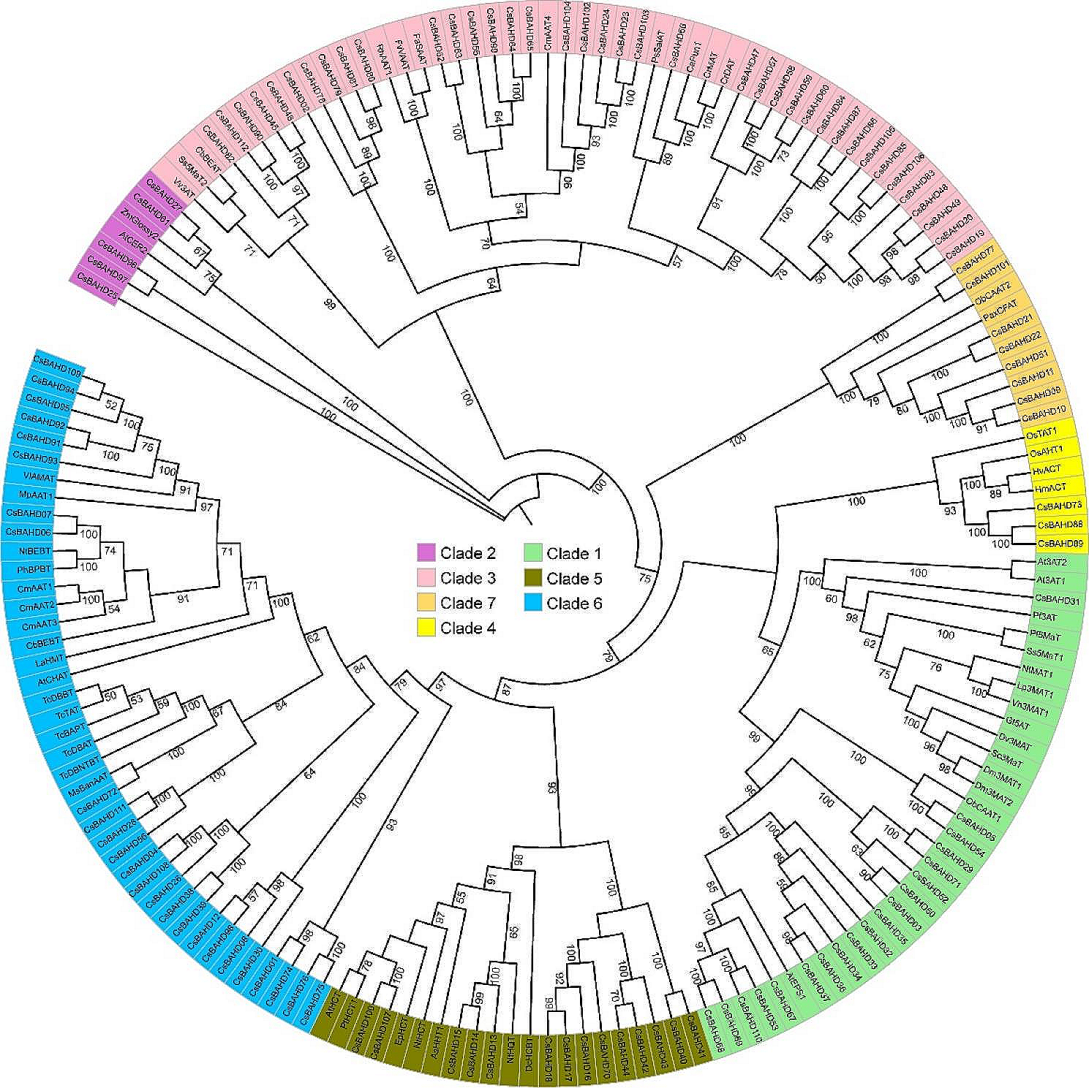
Phylogenetic analysis of the *BAHD* genes in tea plants and other plants. The accession numbers of the other plant BAHD proteins used for the evolutionary tree construction are listed in Table S4

To investigate the evolutionary relationships among members of the tea plant BAHD family, a phylogenetic tree was constructed based on the ML method (Fig. 2). According to the classification criteria of the BAHD gene family in terrestrial plants [3], the 112 CsBAHDs were categorized into seven subgroups (Clade 1 to 7). The number of members in each clade varied dramatically, with 19, 5, 38, 3, 14, 25, and 8 members in Clades 1 to 7, respectively. Notably, in the BAHD clusters CsBAHD13-18, CsBAHD32-37 and CsBAHD91-95 mentioned above, members of the same cluster are clustered in the same clade. For the CsBAHD77-81 cluster, all the members except CsBAHD77 are clustered in Clade 7.

### Gene structure and conserved domain analysis of *CsBAHDs*

**Fig. 3:**
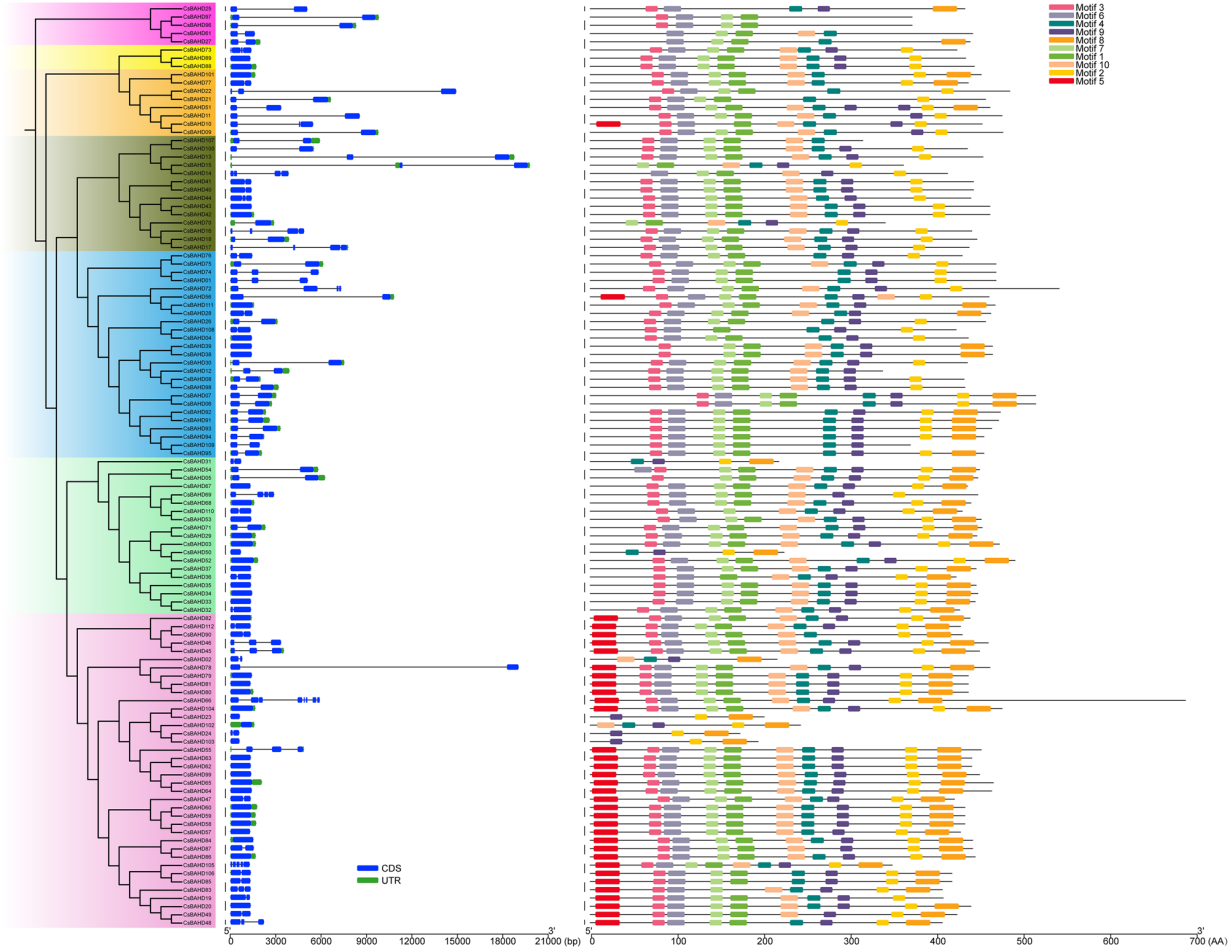
Phylogenetic tree, gene structure and conserved motifs of CsBAHDs

The intron-exon structure analysis of each gene revealed that the number of introns in the *CsBAHDs* ranged from zero to seven (Fig. 3). Other than *CsBAHD66* and *CsBAHD105*, no more than three introns are present in the *CsBAHDs*. Members of the same evolutionary branch exhibited similar gene structures. In addition, the conserved structural domains of the CsBAHD proteins were identified. As shown in Fig. 3, most CsBAHDs in the same clade had a similar number and distribution of Motifs. The members of Clade 2 had the fewest Motif categories, and the members of Clade 3 had the most Motif categories. The two conserved domains HXXXDG and DFGWG are responsible for the catalytic activity and structural integrity of the BAHD family [3, 8] are located in Motif 1 and Motif 2, respectively (Figure S1). The conserved domain NYFGNC associated with flavonoid acylation [4] is located in Motif 9. These three Motifs were respectively absent in 8 (CsBAHD02, CsBAHD23, CsBAHD24, CsBAHD25, CsBAHD31, CsBAHD50, CsBAHD102 and CsBAHD103), 8 (CsBAHD02, CsBAHD12, CsBAHD25, CsBAHD27, CsBAHD61, CsBAHD96, CsBAHD97 and CsBAHD107), and 9 (CsBAHD21, CsBAHD22, CsBAHD27, CsBAHD61, CsBAHD77, CsBAHD96, CsBAHD97, CsBAHD101 and CsBAHD107) CsBAHD family members. With the exceptions of CsBAHD10 and CsBAHD56, Motif 5 was distributed only among the members of Clade 3. However, Motif 8 was absent in both of the former two, whereas all the members of Clade 3 contained it. Moreover, although the conserved domains of most members of the same clade are essentially the same, there are some special members. For example, CsBAHD31 and CsBAHD50 of Clade 1, compared with other members in this Clade, both lack the first five Motifs, which may be related to sequence loss during evolution or incomplete genome annotation.

### Identification of *cis*-elements in the promoters of *CsBAHDs*

**Fig. 4:**
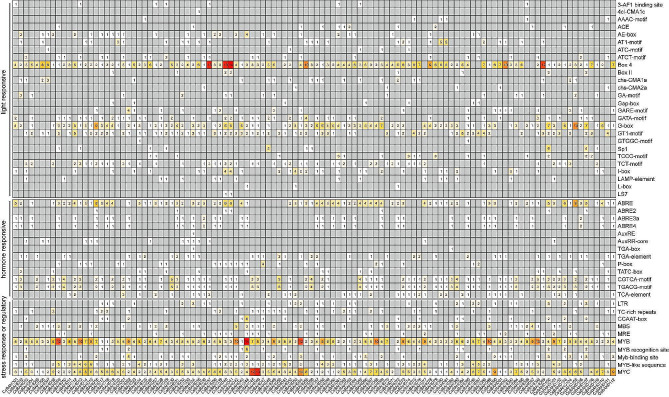
Statistics for the light responsive, hormone responsive and stress-related *cis*-regulatory elements identified in the promoter regions of the *CsBAHD* genes

*Cis*-acting element analysis was performed using 2000 bp upstream of the start codon as the promoter region of the gene. As shown in Fig. 4, a total of 26 types of light-responsive elements were identified, suggesting that the expression of *CsBAHDs* may be strongly influenced by light. Among them, the light-responsive elements Box 4, G-box, and GT1-motif were the most abundant. In addition, we identified 13 types of hormone-related cis-elements, including the abscisic acid (ABA) responsive elements ABRE, ABRE2, ABRE3a and ABRE4; auxin-responsive elements AuxRE, AuxRR-core, TGA-box and TGA-element; gibberellin-responsive elements P-box and TATC-box; MeJA responsive elements CGTCA-motif and TGACG-motif; and the salicylic acid (SA) responsive element TCA-element. Moreover, the low-temperature responsive element LTR, the defense and stress responsive element TC-rich repeats, and different transcription factor binding responsive elements, such as the CCAAT-box, MBS, MRE, MYB, MYB recognition site, Myb-binding site, MYB-like sequence and MYC, were also found. These results suggest that *CsBAHDs* may play an important role in tea plant growth and development and in response to biotic and abiotic stresses.

### Expression analysis of *CsBAHDs*

**Fig. 5:**
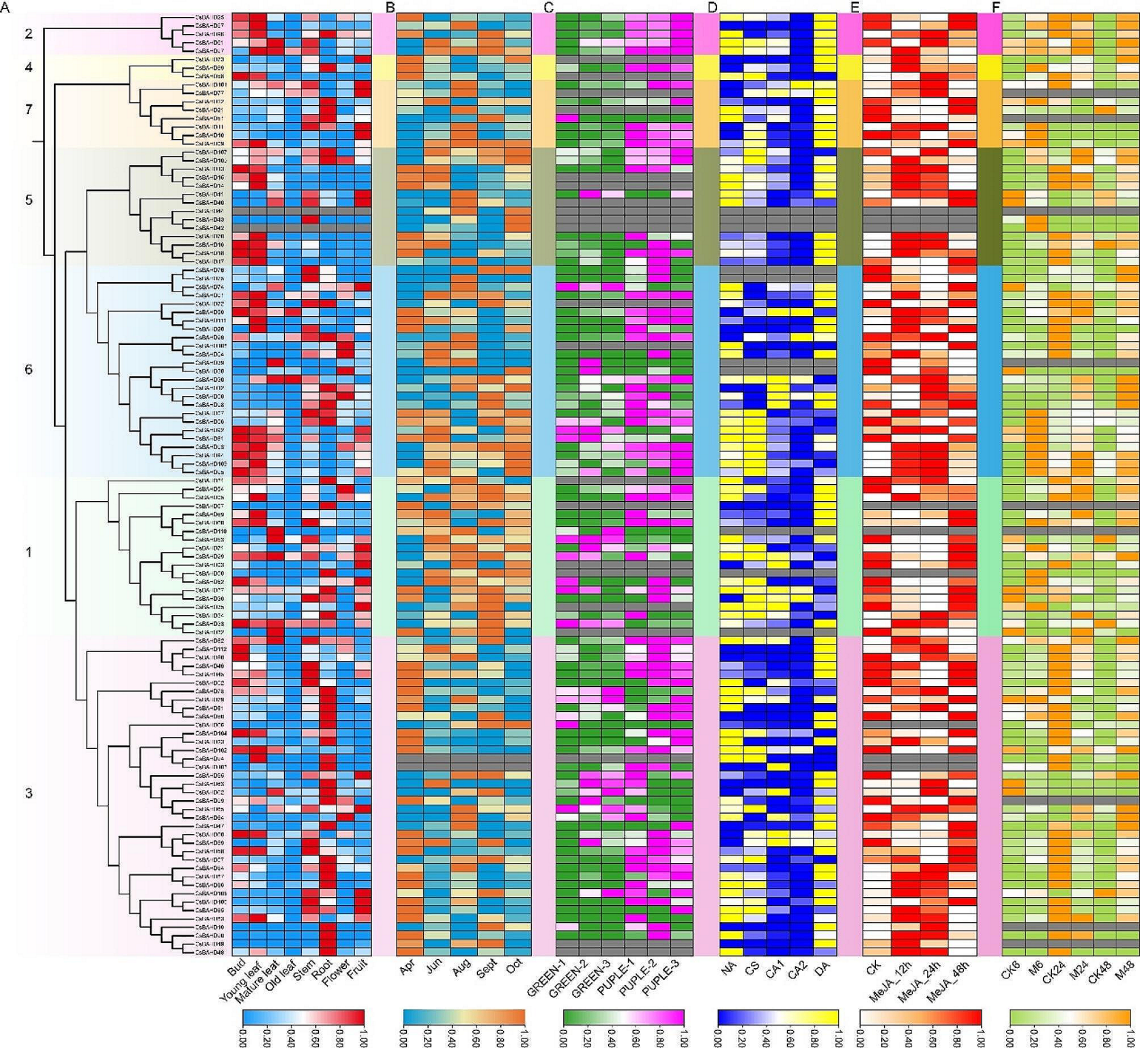
The expression patterns of *CsBAHDs* in tea plants based on public transcriptome data. (A) The expression patterns of *CsBAHDs* in eight different tissues of tea plants. The numbers on the phylogenetic tree represent the seven subgroups in Fig. 2. (B) The expression patterns of *CsBAHDs* in tea leaves from five different months. (C) The expression patterns of *CsBAHDs* in green and purple tea leaves. (D) The expression patterns of *CsBAHDs* in tea plants under cold acclimation. (E) The expression patterns of *CsBAHDs* in tea plants at different time points after MeJA treatment. (F) The expression patterns of *CsBAHDs* in tea plants at different time points after feeding by tea green leafhoppers

To investigate the biological functions of the *BAHD* genes in tea plants, we analyzed the expression of individual members using published transcriptome data. Only two members showed no detectable expression in eight tissues, while the rest showed diverse expression patterns (Fig. 5A). Among them, *CsBAHD93*, *CsBAHD25*, *CsBAHD95, CsBAHD91, CsBAHD60, CsBAHD71, CsBAHD52, CsBAHD01, CsBAHD88* and *CsBAHD94* were expressed mainly (FPKM > 100) in buds; *CsBAHD05, CsBAHD93, CsBAHD25, CsBAHD01, CsBAHD29, CsBAHD60, CsBAHD96* and *CsBAHD91* were expressed mainly in young leaves; *CsBAHD29, CsBAHD30* and *CsBAHD65* were expressed mostly in mature leaves; *CsBAHD30* was expressed majorly in old leaves; *CsBAHD65, CsBAHD71* and *CsBAHD29* were expressed predominantly in stems; *CsBAHD34, CsBAHD57, CsBAHD71, CsBAHD96, CsBAHD84* and *CsBAHD89* were expressed primarily in roots; *CsBAHD05, CsBAHD93* and *CsBAHD54* were expressed mainly in flowers; and *CsBAHD71, CsBAHD03, CsBAHD65, CsBAHD52* and *CsBAHD29* were expressed dominantly in fruits. From the perspective of different months, *CsBAHD05* and *CsBAHD93* exhibited extremely high expression levels (average FPKM > 500) in all five months tested. In addition, *CsBAHD09* was expressed majorly in June, August, September and October; *CsBAHD71* was expressed mainly in June, August and October; *CsBAHD27* and *CsBAHD29* were expressed predominantly in August and September, and *CsBAHD25* and *CsBAHD01* were expressed primarily in April and June, respectively (Fig. 5B).

In view of the functions of the *BAHD* genes in anthocyanin synthesis, we analyzed the expression of *CsBAHDs* in green and purple tea leaves. As shown in Fig. 5C, more than 63% (71/112) of the genes were upregulated in purple leaves. Among these genes, *CsBAHD05* was upregulated more than 500-fold, and *CsBAHD01, CsBAHD96, CsBAHD97, CsBAHD09, CsBAHD27* and *CsBAHD18* were upregulated more than 10-fold.

Since promoter analysis revealed that many *cis*-elements are associated with low-temperature, MeJA and defense and stress responsiveness, we examined the expression of *CsBAHD*s in response to cold acclimation, MeJA treatment and tea green leafhoppers feeding. As shown in Fig. 5D, after low temperature domestication, the expression of most members was downregulated, and that of *CsBAHD05* was downregulated more than 100-fold, while the expression of other members, such as *CsBAHD56* and *CsBAHD30*, was upregulated. In addition, we found that the expression of several genes, such as *CsBAHD93* and *CsBAHD71*, increased in response toshort-term low temperature treatment, and significantly decreased in response to long-term low temperature treatment. After MeJA treatment, we found that the significantly upregulated members were mainly concentrated in subgroups 5 and 6 (Fig. 5E). Among them, *CsBAHD92, CsBAHD93, CsBAHD94, CsBAHD95, CsBAHD109, CsBAHD70* and *CsBAHD16* were most significantly upregulated, especially at 12 and 24 h after treatment. In the tea green leafhoppers feeding treatment, *CsBAHD93, CsBAHD94, CsBAHD95, CsBAHD109* and *CsBAHD07* were the five members with the most upregulated expression at 6 h after feeding, and *CsBAHD93* was upregulated nearly 300-fold (Fig. 5F). *CsBAHD93, CsBAHD107, CsBAHD109, CsBAHD100* and *CsBAHD94* and *CsBAHD05, CsBAHD01, CsBAHD88, CsBAHD60* and *CsBAHD90* were the five members whose expression was mostupregulated at 24 and 48 h after feeding, respectively. These members are well worth following up with functional studies.

### Expression patterns of *CsBAHDs* in response to tea geometrids and tea green leafhoppers feeding

**Fig. 6:**
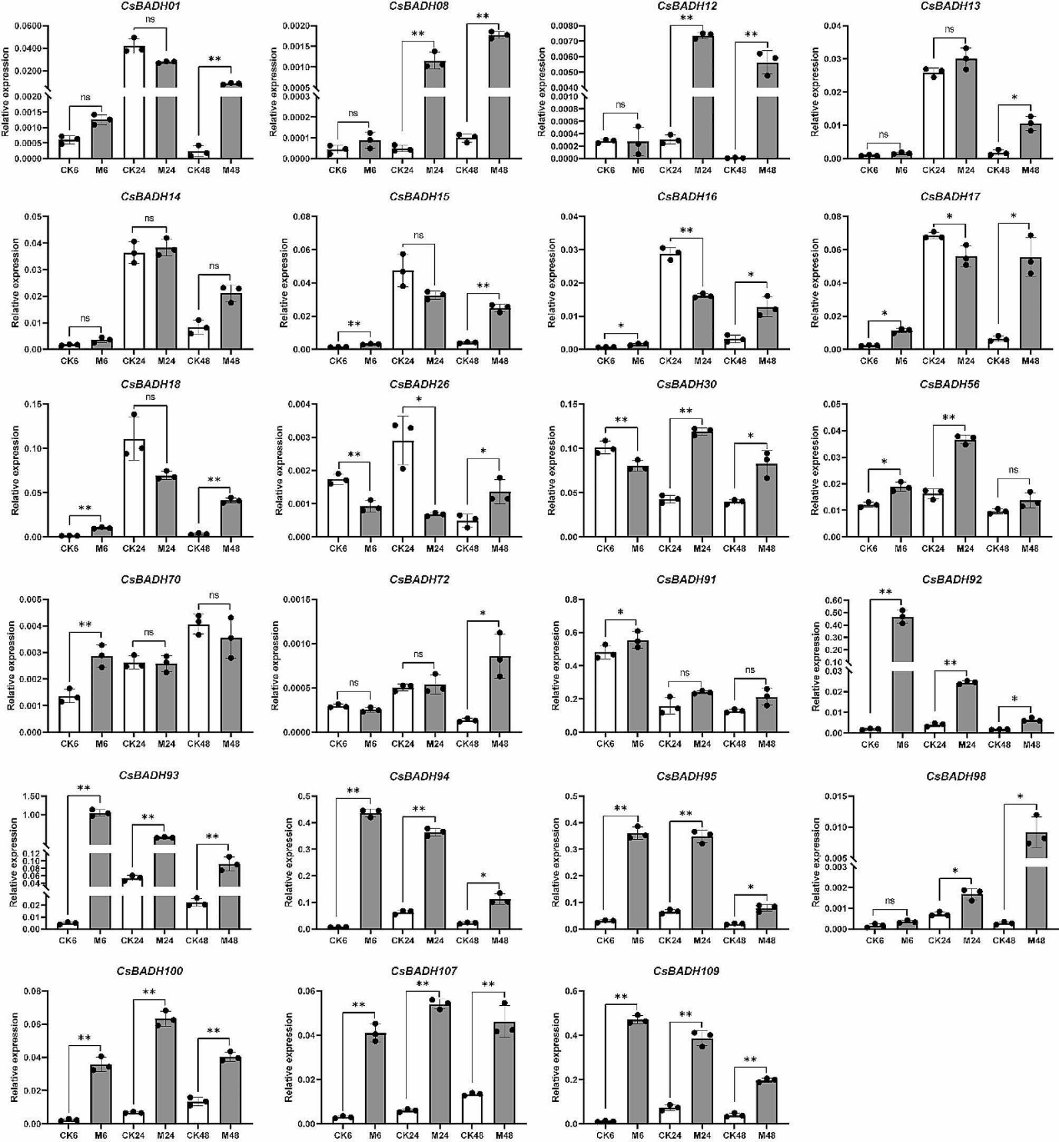
The relative expression levels of the *CsBAHDs* in tea leaves at different time points after feeding by tea green leafhoppers. M6, M24, and M48 represent samples taken 6, 24,48 h after feeding, while CK6, CK24, and CK48 represent control samples at the corresponding time points. The expression levels were calculated based on the 2^−ΔCT^ method relative to the internal reference gene. The bars represent the mean ± SD (*n* = 3). Significant differences between the treatments and CK were determined by Student’s t test (*p < 0.05, **p < 0.01, ns: no significant difference)

**Fig. 7:**
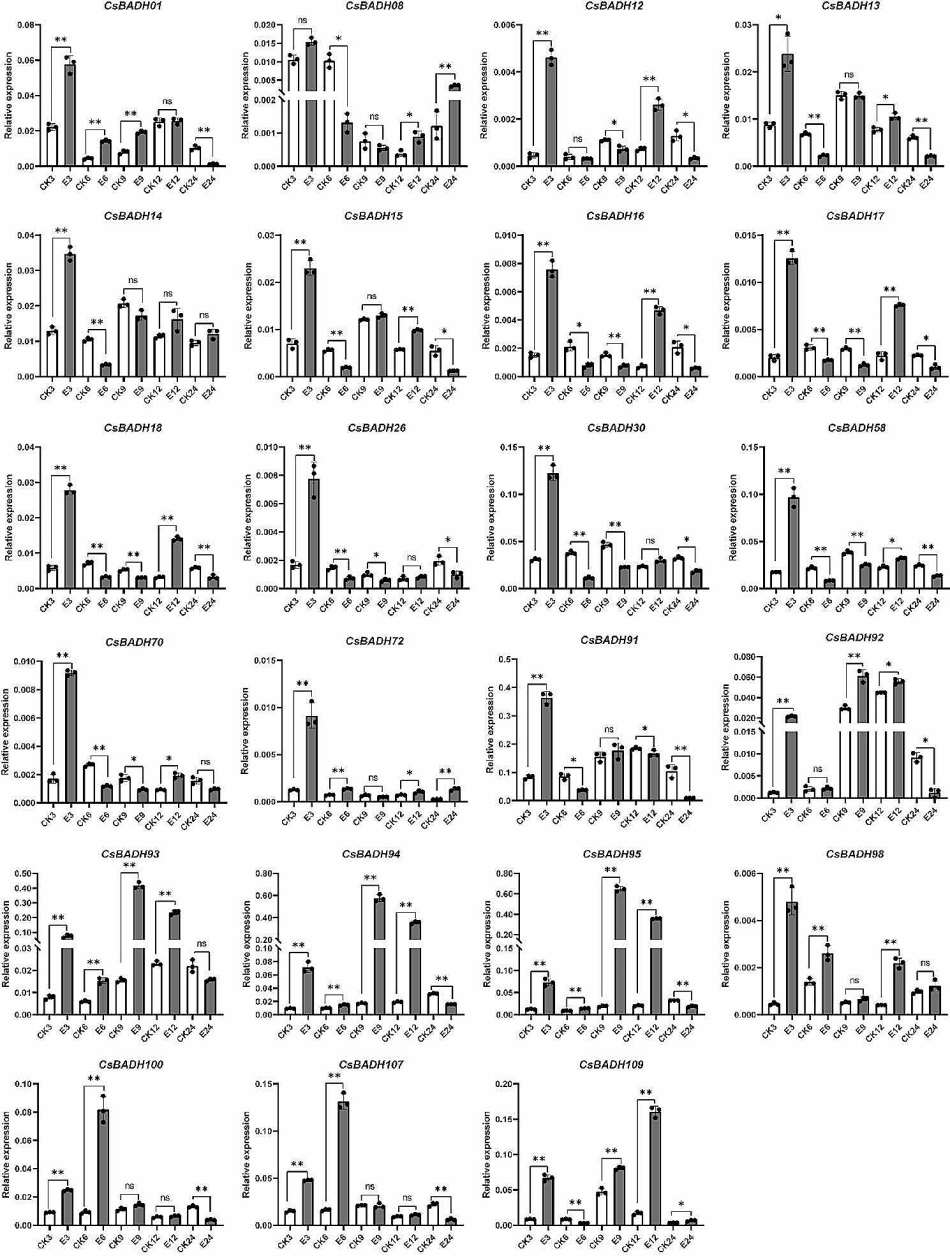
The relative expression levels of the *CsBAHDs* in tea leaves at different time points after feeding by tea geometrids. E3, E6, E9, E12, and E24 represent samples taken 3, 6, 9, 12, and 24 h after feeding, while CK3, CK6, CK9, CK12, and CK24 represent control samples at the corresponding time points. The expression levels were calculated based on the 2^−ΔCT^ method relative to the internal reference gene. The bars represent the mean ± SD (*n* = 3). Significant differences between the treatments and CK were determined by Student’s t test (*p < 0.05, **p < 0.01, ns: no significant difference)

Given that Clade 5 and Clade 6 contain core members of the plant BAHD family and that their products play important roles in pest defense as well as in fruit aroma production [3], we therefore focused on members of these two subgroups in tea plants. We first filtered out members with very low or no detectable expression based on the availability of transcriptome data from tea plants subjected to feeding by tea green leafhoppers and feeding by tea geometrids; ultimately, a total of 23 members were retained for qPCR analysis. As shown in Fig. 6, all genes except *CsBAHD14*were significantly induced at various time points after feeding by tea green leafhoppers. In particular, the expression of *CsBAHD92*, *CsBAHD93*, *CsBAHD94*, *CsBAHD95, CsBAHD100, CsBAHD107* and *CsBAHD109* was significantly induced at all three time points. In addition, although the induced expression levels of *CsBAHD08, CsBAHD12* and *CsBAHD98* did not reach significant levels at 6 h after feeding, they were significantly induced at the latter two time points. For the tea geometrids feeding response, as shown in Fig. 7, we examined the expression of the genes at five time points. Notably, we found that the expression of all 23 genes was significantly upregulated in expression at 3 h after feeding, whereas they subsequently exhibited various expression patterns. Most of them were downregulated at 6 and 9 h after feeding, significantly upregulated at 12 h, and downregulated again at 24 h. *CsBAHD08, CsBAHD72* and *CsBAHD109* were the only three members whose expression was significantly upregulated at 24 h after feeding, while *CsBAHD93*, *CsBAHD94* and *CsBAHD95* were significantly upregulated at the other four time points after feeding.

### Transcriptional regulatory network of *CsBAHDs* in response to tea geometrids and tea green leafhoppers feeding

**Fig. 8:**
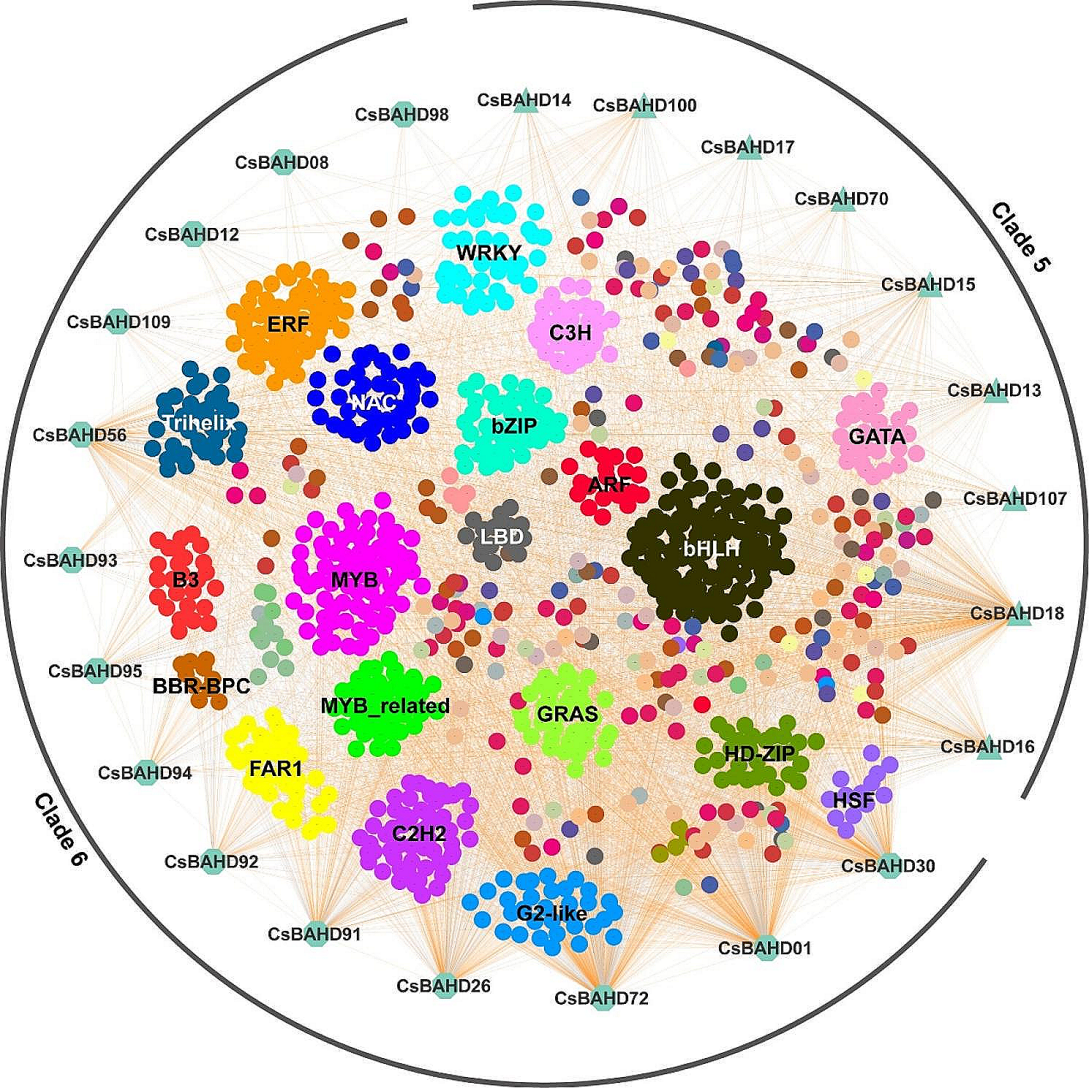
Transcriptional regulation of *CsBAHD* genes in response to feeding by herbivorous pests. The correlations between the expression of *CsBAHD* genes and that of different transcription factors are shown with colored lines (Pearson’s correlation test, *P* ≤ 0.01). The gene correlation expression networks were visualized using Cytoscape v3.7.1 software. The detailed expression correlations used for network construction are listed in Table S6

To further investigate the transcriptional regulation of *CsBAHDs* in response to feeding by tea geometrids and tea green leafhoppers, we screened 1,248 transcription factor (TF) genes from 55 TF families based on the aforementioned transcriptome data and filtering criteria (Table S5), and analyzed the correlation of the expression with the 23 *CsBAHD* genes mentioned above, and constructed a regulatory network based on the significantly correlated members (*P* < 0.01). As shown in Fig. 8, each *CsBAHD* gene had TFs that were significantly correlated withits expression, and the categories of these TFs were diverse. Among them, bHLH, C2H2, MYB, MYB-related, NAC, ERF, WRKY, and bZIP were the most predominant TF families of coexpressed with *CsBAHDs*. These results suggest that the participation of *CsBAHDs* in the feeding response to tea geometrids and tea green leafhoppers may be regulated by different TFs. In addition, we also found significant correlations between the expression of some *CsBAHDs*, indicating that these genes may function synergistically.

## Discussion

In recent years, discoveries about BAHD acyltransferases have been made at an increasing rate, and studies have shown that BAHDs did not originate in plants, but rather pre-date their emergence and that the BAHD family has expanded as plants continued to evolve. Moreover, it is thought that the expansion of BAHDs in plants is related to the adaptation of plants to terrestrial environments [3]. In this study, we identified 112 BAHD family members from the tea plant genome and found that a significant expansion in their number occurred from wild tea plants to large-leaved cultivated tea plants (*assamica* type) to small- and medium-leaved cultivated tea plants (*sinensis* type). This phenomenon is consistent with the domestication of tea plants [47], suggesting that the expansion of BAHD in tea plants is related to the adaptation of tea plants to the environment. This was also found in the previous identification of the ABA receptor protein PYL in tea plants [48]. We found that DSD and TD were the main evolutionary modes of the BAHD in tea plants, which is consistent with what has been observed in *Arabidopsis* and *Populus* [49] but different from that of apple, European pear, Chinese white pear and lavender [10]. It has been suggested that genes involved in stress and specific plant metabolic responses are more likely to undergo lineage-specific diversification through tandem duplication events [50], which further suggests that the expansion of BAHD in tea plants may be related to environmental adaptation during domestication. In addition, the number of BAHDs in the tea plant genome was greater than that in the *Arabidopsis* (61) [49], *Schisandra chinensis* (37) [9], banana (46) [6], black raspberry (69) [8], peach (82) [8], strawberry (89) [8], *Populus* (94) [49] and European pear (97) [8] genomes but less than that in the Chinese white pear (114) [8], sweet cherry (125) [8], apple (141) [8] and lavender (166) [10] genomes. Among them, *Arabidopsis* has the smallest genome (125 Mb) [51], and tea plants have the largest (2.94 Gb) [34], which shows that the number of BAHDs is not directly related to the genome size.

The expansion of the BAHD family in plants was accompanied by functional differentiation, which has been demonstrated in different species. We found that all seven subgroups found in angiosperms were present in tea plants, indicating the diversity of BAHD functions in tea plants. For example, most members of Clade 1 of the BAHD family are involved in anthocyanin/flavonoid/phenolic glucoside metabolism [3]. In this study, we found that a member of this subgroup, *CsBAHD05*, was expressed at more than 500-fold higher levels in purple-leafed tea plants than in green-leafed plants, suggesting that it is a key gene involved in anthocyanin metabolism in tea plants. In addition, anthocyanin metabolism in tea plants is also affected by light and temperature [52], and *CsBAHD05* likewise showed sensitivity to temperature, providing further evidence for its involvement in anthocyanin metabolism. The Clade 2 currently has only three characterized members (AtCER2, ZmGlossy2 and ZmGlossy2-like), all of which are associated with epicuticular waxes synthesis [3]. Epicuticular waxes are essential for plants to cope with environmental stresses [53]. Herein, we identified five members of the tea plant genome whose functions deserve further exploration. The functions of the members of Clade 3 are the most complex [3], and this clade has the highest number of members in tea plants. However, in addition to some tissue-specific expressed members, such as *CsBAHD57*, *CsBAHD60* and *CsBAHD65*, we did not screen for members that are particularly sensitive to stress. A large number of studies have shown that members of Clades 4–7 play an important role in plant responses to adverse stress or in the formation of aromatic volatiles [3]. For example, an acyltransferase (*AT1*) from Clade 4 in tobacco was found to be involved in the synthesis of phenolamides (PAs) as a chemical defense against herbivores [17]. The Clade 6 acyltransferase gene *LoAAT1* can simultaneously synthesize the main aroma components ethyl benzoate and methyl benzoate from *Lilium* oriental flowers [14]. For tea plant, these volatiles not only are major components of tea aroma but also serve as early warning signals to activate the defense response of tea plants [25, 54, 55]. Our transcriptome analyses showed that members with significant stress responses were mostly clustered in these subgroups, especially Clades 5 and 6. We further investigated the feeding response of members of these two subgroups to two main types (pricking and chewing) of herbivorous pests on tea plants by qPCR, and found that *CsBAHD93*, *CsBAHD94* and *CsBAHD95* can significantly respond to feeding by these two types of pests at the same time. Interestingly, these three genes were present in tandem duplicates in the tea plant genome and were more closely evolutionarily related to the *VIAMAT* [56], *MpAAT1* [57], *NtBEBT* [17] and *AtCHAT* [16] genes. These four genes with known functions can all use aromatic alcohols as substrates, which provides ideas for our subsequent exploration of the functions of *CsBAHD93*, *CsBAHD94* and *CsBAHD95*.

In this study, to investigate the biological functions of tea plant BAHDs, we also analyzed the *cis*-acting elements within their promoter regions. We found that they contain a large number of different kinds of light-responsive elements, suggesting that light may affect their function. This is also reflected in the diversity of their expression during different months. A growing number of studies have shown that light regulates the accumulation of secondary metabolites in tea plants [58, 59]. A recent study showed that shading-induced reductions in catechins in tea plant leaves depend mainly on the degree of shading, but the effect of shading on amino acids depends on the season [60]. Moreover, in addition to different stress-associated hormone response elements, a large number of TF binding site elements were also identified, predicting that the functional performance of tea plant *BAHDs* may also be regulated by different TFsas has been found in previous studies. For example, overexpression of a tea plant *BAHD* gene in *Arabidopsis* resulted in significant accumulation of anthocyanin, while many TF genes, such as WRKY, bHLH, and MYB were also significantly upregulated in the transgenic lines [30]. In addition, our correlation analysis further revealed the presence of a large number of TFs coexpressed with different *BAHDs* under tea geometrids or tea green leafhoppers feeding. The mechanism by which TFs regulate the expression of synthetic genes to influence the synthesis of secondary metabolites and thereby regulate the performance of biological functions has been reported in tea plants. For example, *CsMYB42* promoted the expression of *CsGS1c* by binding to the promoter of *CsGS1c*, resulting in high theanine accumulation in albino tea plants [61]. *CsPIFs*-like and *CsMYC2* can able to bind to the promoter of *CsCYP79* and activate its expression to promote increased release of benzyl nitrile to defend against attack by *Ectropis grisescens* [62]. Moreover, in tobacco, *MYB8* controls PA synthesis by regulating the expression of the acyltransferases *AT1*, *DH29*, and *CV86* to modulate the chemical defense of tobacco plants against herbivorous pests [17]. These studies provide an important reference for our subsequent investigation of the mechanism of action of BAHDs in the defense response of tea plants. Our results, in turn, lay the foundation for tsuch investigations.

## Conclusions

In this study, we identified 112 acyltransferase genes (*CsBAHD01*-*CsBAHD112*) from the tea plant genome, with 85% (98/112) distributed across the 15 chromosomes. Tandem duplication and dispersed duplication are the dominant modes of expansion for this family, and a significant expansion in the number of *BAHD* gene family members has occurred from wild tea plants to the *assamica* type to the *sinensis* type. Phylogenetic evolution has shown that all seven clades of this family in angiosperms are present in tea plants, indicating their functional diversity. Promoter *cis*-acting element analysis revealed that the tea plant *BAHD* family contains a large number of light, hormone, and stress-responsive elements. *CsBAHD01, CsBAHD05, CsBAHD25*, *CsBAHD29, CsBAHD52, CsBAHD60, CsBAHD71, CsBAHD88, CsBAHD91, CsBAHD93*, *CsBAHD94*, *CsBAHD95* and *CsBAHD96* were expressed mainly in buds or young leaves. *CsBAHD05* was expressed at more than 500-fold higher levels in purple tea leaves than in green tea leaves. Members that responded significantly to MeJA treatment and herbivorous pest feeding were clustered mainly in subgroups 5 and 6. The members of these two subgroups were verified for different types of herbivorous pest feeding responses using qPCR, and *CsBAHD93*, *CsBAHD94* and *CsBAHD95* were screened to significantly respond to both pricking and chewing pests. They may be involved in the defense response of tea plants through acylation to aromatic alcohols, and this biological function may also be regulated by different TFs. Our results provide a basis for exploring the roles of tea plant BAHDs in tea plant defense responses.

### Electronic supplementary material

Below is the link to the electronic supplementary material.

Supplementary Material 1: **Table S1**. The primers used in this study. **Table S2**. Identification and characterization of the BAHD acyltransferase genes in tea plant genome. **Table S3**. Collinearity of BAHD members in different tea plant genomes. **Table S4**. The accession numbers of other plant BAHD proteins used for the evolutionary tree construction. **Table S5**. Expression level data of BAHD members with different transcription factor genes used for co-expression network construction. **Table S6**. Expression correlation of BAHD members used for co-expression network construction with different transcription factor genes.

Supplementary Material 2: **Figure S1** The conserved motifs in CsBAHDs.

## Data Availability

All data generated or analysed during this study are included in this published article and its supplementary information files.
