# Supplementary figures and images for: Genome-wide identification of tea plant (Camellia sinensis) BAHD acyltransferases reveals their role in response to herbivorous pests
Source: BMC Plant Biol. 2024 Apr 1;24:229. doi: 10.1186/s12870-024-04867-2 (PMC10985903; doi:10.1186/s12870-024-04867-2)

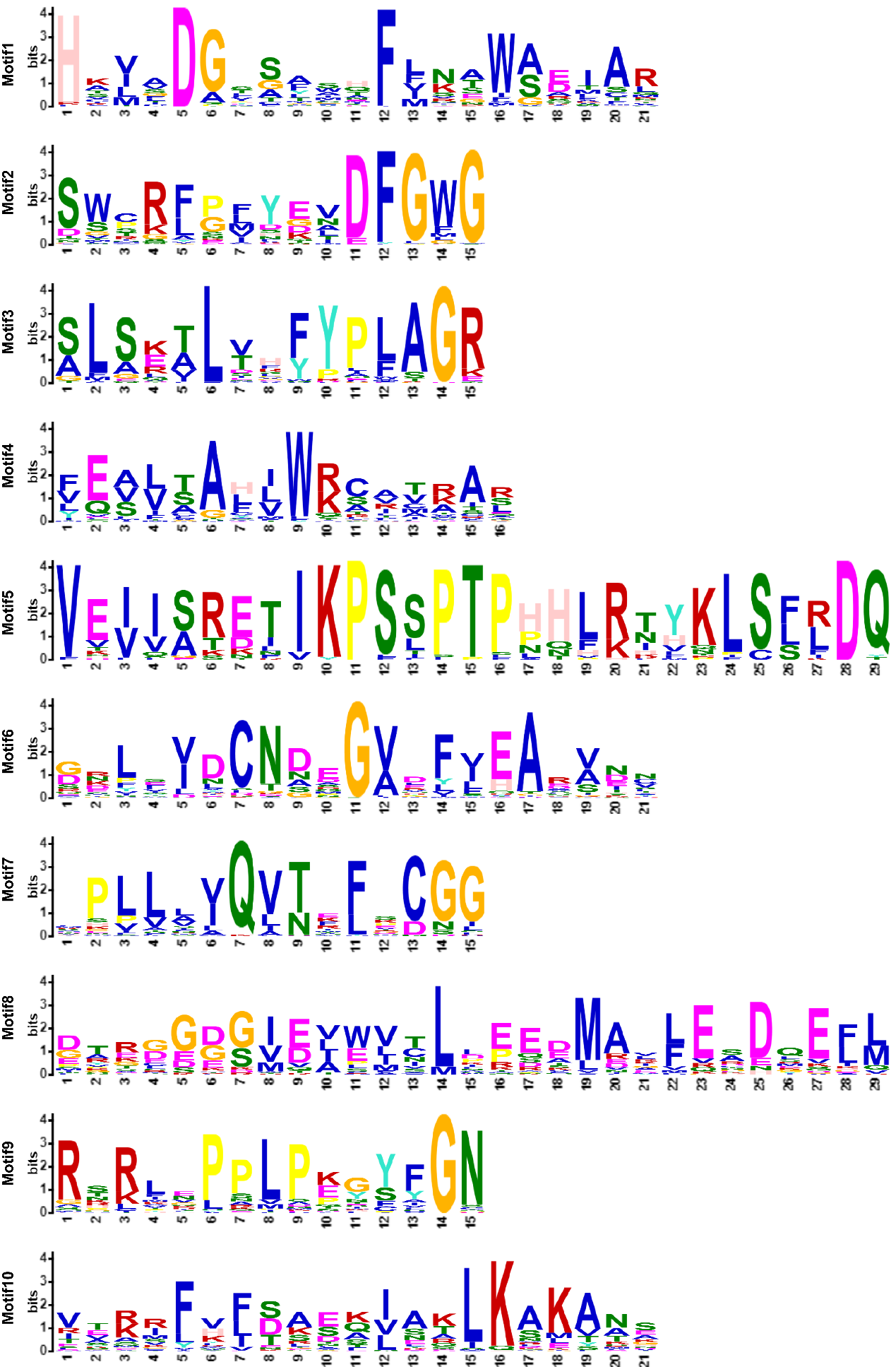


**Figure S1.** The conserved motifs in CsBAHDs.

Supplement: Supplementary file 1 — Supplementary Material 1: Table S1. The primers used in this study. Table S2. Identification and characterization of the BAHD acyltransferase genes in tea plant genome. Table S3. Collinearity of BAHD members in different tea plant genomes. Table S4. The accession numbers of other plant BAHD proteins used for the evolutionary tree construction. Table S5. Expression level data of BAHD members with different transcription factor genes used for co-expression network construction. Table S6. Expression correlation of BAHD members used for co-expression network construction with different transcription factor genes. [file 12870_2024_4867_MOESM1_ESM.docx]
